# Development and validation of a brief screener to measure the Health Literacy Responsiveness of Primary Care Practices (HLPC)

**DOI:** 10.1186/s12875-015-0336-4

**Published:** 2015-09-11

**Authors:** Sibel Vildan Altin, Kristina Lorrek, Stephanie Stock

**Affiliations:** Institute for Health Economics and Clinical Epidemiology, University Hospital of Cologne, Gleuelerstr. 176-178, 50935 Cologne, Germany

## Abstract

**Background:**

The evolving approach of health literate health care organizations (HLHO) receives considerable support from health policy makers. Up to now, there are no performance measures available to assess the application of health literacy strategies by health care professionals in the primary care setting. This paper describes the development and validation of the Health Literate Primary Care Practice screener (HLPC). The screener can be used as a self-assessment tool for primary care organizations (PCO) that aim to elucidate the health literacy responsiveness of their organization.

**Methods:**

The HLPC is a 4-item screener developed in a multi-level process following a theory-driven approach including a literature review, consultations with scientists and cognitive tests with patients in PCO. The screener was applied in a national random sample of *N* = 1125 adults living in Germany. Item and psychometric properties were analyzed by determining item discrimination and reliability as well as performing a confirmatory factor analysis (CFA) to test the instruments unidimensionality. Criterion validity was investigated by performing bivariate correlations between the HLPC score and heath care quality measures.

**Results:**

The HLPC scale demonstrated good item discrimination and internal consistency (α = 0.86). CFA verified a one-factor structure of the scale and analysis on the criterion validity revealed a significant correlation between the HLPC score and patients satisfaction with the general practitioner, accessibility of the PCO and support in care-coordination received in the PCO.

**Conclusions:**

The HLPC is a valid screener to provide insights in the extent of the utilization of health literacy strategies in primary care practices.

## Background

Health care systems increasingly face a rapid transformation of care processes and structures that arise from fast-moving technological developments in the fields of medical research and health information technology resulting in advances in health care coverage and delivery system design [1]. These changes constantly increase the complexity of health care systems and demand more health literacy skills denoted as the capacity to obtain, process and understand basic health information and services to make appropriate health decisions and effectively navigate the health care system [2–4]. Unfortunately, a large part of the US and European population is not sufficiently equipped with these skills leading to undesirable outcomes including a limited adherence to medication-regimes (e.g. patients with coronary heart disease, HIV, elderly populations) and insufficient self-management skills (e.g. patients with diabetes, health failure, coronary heart disease) as well as more frequent hospitalizations and emergency care utilization (e.g. patients with asthma, coronary heart disease, heart failure, older populations [5–7]. Consequently scholars and policy makers propose to address the challenges of limited health literacy by transforming health care organizations to more health literacy responsive ones, arguing that these are responsible for delivering care in a way that it does not require advanced health literacy skills of the patients [8, 9]. According to the Institute of Medicine (IOM) and its corresponding report on the “10 attributes of health literate health care organizations” framework a health literacy responsive organization is one that supports low literate patients to navigate, understand, and use information and services to take care of their health [10]. Crucial elements of such organizations are the application of patient-centred care and the use of appropriate health literacy strategies [11]. The policy related objective of this approach is to integrate the health literacy responsiveness of health care systems and organizations into health care performance measurement in inpatient and outpatient care [12]. This can be achieved by developing comprehensive interventions and matching assessment tools to gauge the health literacy responsiveness of health care systems.

By now, the number of health literacy intervention-toolkits for health care organizations is increasing whereas instruments that assess the extent of the health literacy responsiveness in health care organizations are scarce, particularly with regard to primary care [13–16]. According to health care decision-makers and scholars, health literacy friendly structures and processes are especially relevant in primary care, which cover large parts of the medical routine care and coordinate care for vulnerable populations, such as chronically ill patients [17, 18]. Therefore, there is an increasing need for feasible performance measures assessing the health literacy responsiveness in primary care from the patients perspective [15]. In this regard, academic literature reveals that it may be promising to focus on the measurement of the actual application of health literacy skills in health care organizations, such as skills in interpersonal communication and navigation assistance [10, 15, 19]. However, patient-reported measures assessing the application of these health literacy skills by health care professionals in the primary care setting are scarce [15].

This paper describes the development and validation of the Health Literate Primary Care Practice screener (HLPC) using a multi-level process and a theory-driven approach following the theoretical framework of the IOM framework of a health literate health care organization. The screener was designed for application in a national survey of the German adult population and serves as a performance measure for the assessment of the health literacy responsiveness of primary care practices focusing on the application of health literacy strategies through the health care workforce in the fields of interpersonal communication and navigation assistance.

## Methods

### Instrument development

The HLPC screener was developed to assess the health literacy responsiveness of primary care practices denoted as their capacity to help patients to better navigate the health care system and understand as well as use information and services to take care of his health by using strategies in interpersonal communication and navigation assistance. The screener was developed based on the findings of a literature review on available validated instruments that aim to determine patients health literacy related communication and navigation assistance experiences in health care delivery in the primary care practice. The literature review resulted in the identification of valid instruments that measure the attributes of health literate health care organizations regarding communication in the primary care practice [10, 15, 20, 21]. Since our purpose was to focus more on the aspect of health literate provider communication in the primary care practice, we focused more on the attributes 6 and 7 of the IOM framework, that deal with the actual application of health literacy skills through the organizational workforce [10]. Following this approach, we prioritized measurement instruments that place a great emphasis on the health literacy related aspects of interpersonal communication and navigation assistance and allow an assessment from the patients perspective. Consequently, we identified two instruments appropriate for the primary care practice setting that suited our purpose. The *Consumer Assessment of Healthcare Provider and Systems* (CAHPS) clinician and group survey developed by the Agency for Healthcare Research and Quality (AHRQ) is an assessment tool specifically developed to gauge patients opinions on provider’s use of health literacy communication-strategies in the outpatient setting. The validated 15 item version of the CAHPS (6-items provider communication) is built upon a rigorous development process, including an environmental scan to identify item domains, consultation with experts, item drafting, cognitive testing and psychometric analysis [20]. It was used in a variety of health care settings [22–25]. We adapted the provider communication items and translated them in German by following the guidelines for translating CAHPS surveys [26]. The *Communication Assessment Tool* (CAT) is a reliable and valid 15-item questionnaire that measures interpersonal and communication skills of health care providers from the patients perspective [21]. The CAT was also translated into German. To assess the quality of both instruments three researchers independently reviewed the communication items used in both instruments applying the standardized COSMIN checklist developed to assess the methodological quality of studies reporting measurement properties of measurement tools [27]. The COSMIN checklist is used to assess the reliability and interpretability of measurement properties by reviewing descriptive (i.e. missing values, floor and ceiling effects, item difficulty) and psychometric instrument parameters such as internal consistency, reliability, measurement error, content validity (i.e. .face validity), construct validity (i.e., structural validity, hypotheses testing, and cross-cultural validity), criterion validity, and item responsiveness. The researchers obtained the required information on the methodological quality of the instruments and judged the quality. The methodological quality of both instruments was satisfactory. After the quality assessment the items were pretested in five cognitive interviews using the cognitive technique of probing with 2 male and 3 female patients (≥40 years) frequently attending health care in the primary care setting. The sample size of five interviews was determined by the point at which data saturation occurred, that is, the interpretations were consistent and appropriate, and no new critical difficulties emerged [28]. The method of cognitive interviewing is a standard diagnostic tool for pre-testing survey instruments such as questionnaires and has become increasingly wide spread in social sciences [29]. It is derived from social and cognitive psychology and enables to explore the processes by which respondents answer survey items, and the factors which influence the answers they provide [30]. The participants of the interviews read each item and rated the items with regard to comprehensibility and item relevance in regard to health literate primary care practices using two probing questions per item. For this purpose, the participants were asked to report all their thoughts after reading each item, especially concerning item comprehensibility and judge the importance of each item on a 5-point Likert scale from “1 = not important” to 5 = very important” for a health literate primary care practice in regard to interpersonal communication and navigation assistance. Participants were recruited by distributing information leaflets on the study in 10 primary care practices (one practice per district) in Cologne, Germany. This pretest of all communication items used in both instruments resulted in the selection of 4 items for the HLPC found to be easy to understand and very important for a health literate primary care practice.

### Data collection and sample

The 2013 Commonwealth Fund International Health Policy Survey of the German general population involved computer-assisted telephone interviews with a random sample of adults aged 18 and older living in Germany. The survey was carried out by the survey company Social Science Research Solutions (SSRS) and the BQS Institute for Quality and Safety. The sample was contacted from February to May 2013 by random-digit dialing of both landlines and mobile phones covering whole Germany. Up to eight calls were made to establish contact. Interviewers ascertained whether there were residents in the household within the age range and, if there were multiple, they selected one for the interview using the nearest birthday method. The margin sample error was approximately plus or minus 3 percent for the sample.

Ethical approval from the Regional Ethical Review Board was not required, since the survey was non-medical. Participation in the survey was voluntary. Written informed consent was obtained from every participant before the questionnaire was answered. Confidentiality was maintained by data coding to eliminate the identification of data with personal information.

### Measures

#### HLPC scale

HLPC items were derived from two validated instruments (CAHPS; CAT) used for the assessment of health care provider health literacy communication skills [20, 21]. Instrument items that were included had to undergo a rigorous assessment by social science researchers and a rating process by patients in five cognitive interviews. Each of the final HLPC items is answered on a 4-point Likert scale ranging from “always” to “never”.

#### Experiences with health care delivery in the primary care setting

In order to assess criterion validity several additional measures were developed serving as criterions. Since health literate organizations aim to help patients to “better navigate the health care system and understand as well as use information and services to take care of his health” [10], we assessed whether there is a strong association between the HLPC score and heath care quality measures using three indicators. We measured respondents perceived 1) satisfaction with the own general practitioner in the last 12 month, 2) accessibility of the primary care practice as well as 3) help in care-coordination received by the general practitioner. The indicators were derived from the health care system performance framework of the Commonwealth Fund (CWF) [31] and were developed for the longitudinal Health Policy Survey of the Commonwealth Funds. The CWF framework encompasses 37 indicators within five sections: healthy lives, quality, access, efficiency, and equity of the health care system. For our purpose, we used indicators measuring the performance of a health care system with regard to health care delivery, focusing on health care coordination in ambulatory care, access to health care and satisfaction with health care. Satisfaction with the general practitioner was measured using the item “How do you rate the overall medical care received in the last 12 months by your general practitioner?”. Response was assessed on a 4-point Likert scale ranging from “1 = poor” to “4 = very good”. Perceived accessibility of the primary care practice was assessed asking “If you call your general practitioner with a medical problem during office hours, how often do you receive an answer on the same day?”. Response was assessed on a 4-point Likert scale ranging from “1 = never” to “4 = always”. Perceived help in care coordination was measured asking “How often does your GP help you coordinate or arrange the care you receive from other doctors and places? (for instance making appointments, following up your treatment)”. Response was assessed on a 4-point Likert scale ranging from “1 = never” to “4 = always”.

Support in care-coordination and a good accessibility of the primary care practice are pivotal for navigation purposes [18, 32]. In this regard, advanced care-coordination is considered as a significant component of a health literate health care organization that can help to improve care especially for less health literate patients [33, 34]. In addition, a good accessibility of primary care determines the equity of health care systems and is pivotal for vulnerable populations, such as patients with chronic conditions, increased healthcare needs and limited health literacy skills [18]. By considering these findings, practice accessibility and support in care-coordination should be secured in a health literate health care organization. Therefore, we assumed that respondents who assess the accessibility of their primary care practice as being rather good and perceive greater support by the general practitioner in the coordination of their care would rate their primary care practice more health literacy responsive on the HLPC scale.

Interventions on health literacy and patient-centered care aiming to improve interpersonal communication between general practitioners and patients demonstrate increased patient satisfaction with care and further studies reveal a rather positive association between a higher health literacy level and patients satisfaction with care in the primary care practice [32, 35]. Therefore, we hypothesized a positive association between a patients overall satisfaction with the general practitioner and the perceived health literacy responsiveness of the primary care setting measured on the HLPC scale.

### Data analysis

Descriptive statistics was generated for each item to determine the extent of missing values and floor and ceiling effects. To establish psychometric properties of the HLPC, classical test theory was employed gauging item difficulty and discrimination as well as scale reliability and validity.

Cronbach α coefficient was calculated to determine the internal consistency of the scale. A value of α ≥ 0.7 was considered acceptable, α ≥ 0.8 good and α ≥ 0.9 excellent [36, 37]. Internal consistency was also assessed by item-total correlation, measuring the strengths of the association between an individual item and the total score. A correlation coefficient of 0.30 or higher is recommended in the literature [38].

Explanatory factor analysis (EFA) with the WLSMV estimation method and varimax rotation was conducted to confirm the dominant latent factor and a confirmatory factor analysis (CFA) was performed to further test the underlying construct identified from the EFA. Statistical evidence for unidimensionality was established by determining the goodness of fit indices of a one-factor CFA model. Goodness of fit was tested using several fit indices, such as the root mean square error of approximation (RMSEA), the goodness-of-fit (CFI), the ration of maximum likelihood *x*^2^ to the degrees of freedom (*x*^2^/d.f.), the Tucker-Lewis Index (TLI) and the Weighted root mean square residual (WRMR). When assessing the model fit we considered recommended thresholds of central goodness of fit measures [39–41].

Criterion validity was assessed by exploring the association of the scale score with the overall patient satisfaction with the general practitioner, the extent of assistance in care-coordination by the general practitioner as well as the accessibility of the primary care practice. It was hypothesized, that a perceived higher health literacy friendliness of the primary care practice might be associated with a higher overall satisfaction of the patient with the general practitioner as well as a stronger involvement of the general practitioner in care coordination and a better accessibility of the primary care practice. For that purpose, bivariate correlations were calculated using Spearman’s rank order correlation (rho).

Cases with more than 30 % missing values in the HLPC score and more than 5 % missing values per item were excluded from the analysis. The final sample was weighted to reflect the distribution of the adult German population. Descriptive statistics and bivariate correlations were calculated using IBM SPSS 22.0 including AMOS 22.0 and the EFA and CFA were conducted using MPlus 6.

## Results

### Participants

The overall response rate of the survey is 11.0 %, defined as completed interviews (*N* = 1125) out of the overall sample members that could be contacted (*N* = 10.300). The considerably low response rate indicates a non-response bias.

Characteristics of our survey sample are described in Table 1. Survey participants are in average 52.4 years old, 60 % are female and 43.6 % have a high school education or less. Health status variables indicate that, in general, the sample has good to very good health and only a small group of the sample is affected by chronic conditions and multimorbidity. Nearly all respondents, (94.8 %) do have access to a primary care practice they consult on a regular basis.

**Table 1:** Demographic characteristics

Variable	N	%
Participants	1125	
*Age*		
Mean ± SD	52.4 ± 17.7	
Range	18-96	
*Gender*		
Female	680	60.4
Male	445	39.6
*Migration status*		
Non-migrant	921	82.4
Migrant	197	17.6
*Education degree*		
Middle school degree	192	17.1
Intermediate high school degree	298	26.5
University entrance qualification	254	22.6
University degree	195	17.3
*Living environment*		
Urban	504	44.8
Rural	607	54.0
*Insurance status*		
Statutory health insurance	963	85.6
Private insurance	151	13.4
General practitioner as regular doctor	1066	94.8
*Overall health status*		
Very good	408	36.3
Good	444	39,5
Fair	202	18.0
Poor	61	5.4
*Chronic conditions*		
Diabetes	99	8.8
Coronary artery disease	143	12.7
Hypertension	349	31.0
Asthma	114	10.1
Depression	134	11.9
Multimorbidity (>3 chronic conditions)	168	16.0

### Psychometric properties

The levels of missing data and descriptive statistics for the items are presented in Table 2. Missing values per item did not exceed 5 %. Mean scores for single items were generally skewed towards positive ratings ranging from 3.56 to 3.73. The discrimination was measured with corrected item-total correlations. All item-total correlation coefficients exceeded the 0.4 criterion ranging from 0.54 to 0.69, which indicates very good discrimination. The HLPC scale demonstrated good internal consistency, with a Cronbach’s alpha of 0.86. Together, these findings suggest satisfactory internal reliability.

**Table 2:** Descriptive statistics; factor loadings and internal consistency

		All participants (*N* = 1125)					
No.	Item	Missing (%)	Skewness	Item difficulty (Mean^a^, SD)	Discrimination^b^	Factor Loadings	R^2^
1	When you need care or treatment, how often does your general practitioner or medical staff you see know important information about your medical history?	5	−2,479	3.69 (0.72)	0.58	0.65	0.42
2	When you need care or treatment, how often does your general practitioner or medical staff you see spend enough time with you?	5	−1,891	3.56 (0.82)	0.68	0.76	0.58
3	When you need care or treatment, how often does your general practitioner or medical staff you see encourage you to ask questions?	5	−2,059	3.59 (0.83)	0.69	0.78	0.61
4	When you need care or treatment, how often does your general practitioner or medical staff you see explain things in a way that is easy to understand?	5	−2,610	3.73 (0.64)	0.54	0.71	0.50

^a^Items are scored 1–4; ^b^Cronbach’s α of corrected Item-total correlation

### Confirmatory factor analysis

Explanatory factor analysis (EFA) with the WLSMV estimation method and varimax rotation revealed a one-factor structure explaining 52.4 % of the total variance. Confirmatory factor analysis of a one-factor model showed good fit resulting in a model with x^2^/d.f. [10.413/16] = 0.651; *p* < 0.005; RMSEA = 0.063; TLI = 0.973; CFI = 0.991; WRMR = 0.570 as presented in Table 3. All fit indices met the predefined thresholds demonstrating that the 4 items contribute to a total measure of HLPC (unidimensionality). The factor loadings in the one-factor model ranged from 0.65 to 0.78 as presented in Table 1.

**Table 3:** Measures of global fit confirmatory factor analysis

	x^2^	d.f.	p	x^2^/d.f.	TLI	CFI	RMSEA	WRMR
Thresholds for acceptable fit				≤2	≥0.95	≥0.95	≤0.08	<1.0
One-factor model	10.413	16	<0.005	0.651	0.973	0.991	0.063	0.570

*CFI* comparative fit index; *RMSEA* root mean square error of approxamination; *TLI* Tucker-Lewis Index; *WRMR* weighted root mean square residual

### Criterion validity

Table 4 gives the results for criterion validity testing. The correlations between the HLPC scale score and other single items were significant at the *p* < 0.01 level. The correlation was strongest for the HLPC scale score and satisfaction with the GP in the last 12 month, resulting in a correlation coefficient of 0.47. Perceived help in care coordination by the GP and accessibility of the GP were moderately correlated to the HLPC scale score with coefficients of 0.28 and 0.25, significant at the p < 0.01 level.

**Table 4:** Correlations between scale score and individual items

Scale/item	Scale score
	*N* = 1025
Satisfaftion with GP	0.466
Coordination of care by the GP	0.278
Accessability of GP	0.251

*All bivariate correlations are significant at *p* < 0.01

## Discussion

This study was part of the 2013 Commonwealth Fund International Health Policy Survey on a random sample of 1125 adults aged 18 and older living in Germany. The aim was to assess experiences with health care delivery in outpatient and inpatient care from the patient perspective with special emphasis on the perceived health literacy friendliness and patient-centeredness in the primary care setting [42]. The development of the HLPC followed a thorough multi-level process. It included two reviews of the literature regarding (1) conceptual characteristics of a health literate health care organization and (2) available validated instruments that aim to determine patients health literacy related experiences in health care delivery in the primary care practice. This review resulted in the identification of the “IOM 10 attributes framework” and the selection, translation, thorough evaluation (consultation with social scientists, cognitive testing with patients in the primary care setting) and adaptation of valuable and comprehensive tools that measure the IOM framework [10, 20, 21]. When selecting suitable tools we certainly focused on instruments that measured the actual application of health literacy skills by the workforce in primary care practices, prioritizing aspects of interpersonal communication and navigation assistance, as recommended in earlier studies [11, 12]. In this regard, the HLPC mainly addresses the organizational health literacy aspects of interpersonal communication and navigation assistance in primary care allowing primary care practices to investigate the extent to which their communication and navigation services meet the informational needs of patients and take action where applicable. According to Brach and colleagues who developed the “10 attributes framework” for health literate health care organizations, the application of health literacy strategies in interpersonal communication including the confirmation of understanding (attribute six and seven in the framework) has the far most direct relevance to health care professionals working in health literate health care organizations [10]. Therefore, the HLPC could substantially contribute to establish a brief self-assessment tool for primary health care organizations that aim to elucidate the status quo in their organization and seek guidance in matters of health literacy related skills for their health care workforce.

The HLPC scale underwent a thorough process of piloting and testing for unidimensionality, internal reliability and factor as well as criterion validity. Evidence for internal consistency, reliability and criterion validity obtained was satisfactory, indicating that the HLPC can be considered a high-quality instrument for the primary care setting. The results of the EFA and CFA were supportive to determine the scale structure and the evidence of criterion validity helped to quantify the significance of the scale for perceived health care quality in terms of primary practice accessibility, provision of coordination assistance and overall satisfaction with the general practitioner. This results support the notion that the HLPC could be used as an additional indicator of health care quality on a national level elucidating the importance of health literacy friendliness in primary health care organizations.

The main strengths of this national study is the use of representative data of the German general population obtained by generating a random sample by applying random-digit dialing of both landlines and mobile phones and weighing the sample to reflect the distribution of the adult population in Germany. In addition, the development of the HLPC screener was a multi-level process following a theory-driven approach and performing the validation thoroughly.

However, some limitations of the study should be considered. It should be taken into account that the HLPC does not measure the whole continuum of the 10 attributes of a health literate health care organization, as proposed by the IOM. It rather prioritizes the actual application of health literacy skills, such as supportive communication and navigation assistance representing attribute 6 and 7 of the IOM framework [10]. According to these attributes health literate organizations meet the needs of populations with a range of health literacy skills by avoiding stigmatization and applying health literacy strategies in interpersonal communication and at all points of contact. Since the actual application of health literacy skills in direct communication and care coordination assistance are most relevant to patients with limited health literacy skills when seeking medical care, the HLPC covers crucial components of health literate health care organizations [11]. From a methodological perspective, it should be mentioned that a major weakness of this study is the considerably low response rate of 11.0 % increasing the potential of a non-response bias. In this regard, adults with low literacy levels may try to hide their difficulties [43] and therefore be less likely to respond to surveys [44]. In addition, people with poor literacy, low socio-economic status, members of minority ethnic groups and the elderly are recognized as groups which are hard to reach in social science research [45, 46]. Therefore, there is potential that our study overlooked especially these groups. One possible reason for the high non-response might be the rapid response design of the survey with a field time of eight weeks. However, it needs to be pointed out, that interviewers called potential survey participants at least eight times if they did not receive a response. Consistent with previous findings, some skewing towards positive assessment occurred among all four items [20, 24]. Whether this reflects positive experiences or low expectations is currently unclear. Further studies should go further in validating the HLPC, for example with regard to retest-reliability, predictive and construct validity. Beyond that, standard values will have to be established.

## Conclusion

The development of valid and reliable instruments for the measurement of patients experiences regarding the health literacy friendliness of primary care practices is pivotal in terms of quality assurance efforts and patient-centeredness matters. The HLPC is an appropriate instrument to provide insights in the extent of the utilization of health literacy strategies in primary care practices and offers organizations a feasible and reliable opportunity to obtain a clear picture on the health literacy friendliness of their organization from the patients point of view. The present study has provided strong evidence for the internal consistency, reliability and criterion validity of the HLPC. Further investigations of the construct and predictive validity of the HLPC are warranted.

## Abbreviations

HLPC: Health literate primary care practice screener
HLHO: Health literate health care organizations
PCO: Primary care organizations
IOM: Institute of medicine
CAHPS: Consumer assessment of health care provider and systems
AHRQ: Agency for healthcare research and quality
CAT: Communication assessment tool
SSRS: Social science research solutions
BQS: Institute for quality and safety
CFA: Confirmatory factor analysis
EFA: Explanatory factor analysis
WLSMV: Estimation method and varimax rotation
RMSEA: Root mean square error of approximation
CFI: Goodness-of-fit
TLI: Tucker-Lewis index
